# *Cell Division*, a new open access online forum for and from the cell cycle community

**DOI:** 10.1186/1747-1028-1-1

**Published:** 2006-04-03

**Authors:** Philipp Kaldis, Michele Pagano

**Affiliations:** 1 National Cancer Institute, Mouse Cancer Genetics Program, Bldg. 560/22–56, 1050 Boyles Street, Frederick, MD 21702–1201, USA; 2 Department of Pathology, NYU Cancer Institute, New York University School of Medicine, 550 First Avenue, MSB 599, New York, NY 10016, USA

## Abstract

*Cell Division *is a new, open access, peer-reviewed online journal that publishes cutting-edge articles, commentaries and reviews on all exciting aspects of cell cycle control in eukaryotes. A major goal of this new journal is to publish timely and significant studies on the aberrations of the cell cycle network that occur in cancer and other diseases.

Eukaryotic cells undergo cell division to produce two daughter cells with a DNA content identical to the parental cell. This process is mediated by a complex, multimeric machine composed of an effector protein-phosphorylation engine which is itself controlled and governed by a regulatory protein network. Activation of the phosphorylation engine by cyclin-dependent kinases (CDKs) drives the transitions between the different phases of the cell cycle. Various regulatory proteins provide a precise framework for the regulation of CDK activities. The cell cycle clock is turned on and off in response to mitogenic and anti-mitogenic stimuli that initiate various signal transduction events and ultimately impinge on the activity of the CDKs. In addition, a network of surveillance mechanisms or checkpoints is responsible for the correct order and unidirectional nature of the cell cycle.

The Cell Cycle research field has matured and grown over the past two decades, culminating in the 2001 Nobel Prize awarded to Leland Hartwell, Tim Hunt and Paul Nurse. The precise and accurate regulation of the cell cycle is essential for cellular division in the context of normal development. Alterations in cell cycle mechanisms have been shown to contribute to unrestricted proliferation and oncogenic transformation. A major challenge for the field is a broader use of multiple model organisms in vivo. This is in addition to the challenges of studying cell cycle regulation in cell culture. Overcoming these challenges will allow a deeper understanding of the cell cycle, help to identify potential cancer targets, and promote the development of novel strategies for cancer therapy.

In sum, the eukaryotic Cell Cycle field comprises a number of related topics ranging from the signal transduction pathway to gene expression, from DNA replication to DNA repair, and from checkpoints to finally cancer biology.

The editorial board of *Cell Division *[1] includes 40 associate editors all of whom are leaders in the field of Cell Cycle regulation [2]. As a logo for the journal, we have chosen the MoebiusII strip. This logo represents a DNA molecule folding upon itself and is meant to embody the infinite and cyclic nature of life. We consider this logo to be appropriate for a journal that aims at studying and deciphering the mechanisms regulating the cycle that generates new cells.

*Cell Division *[1] aims to be an open access, online forum for and from the cell cycle community. The purpose of the journal is to publish cutting-edge aspects of cell cycle research and to bridge the gap between models of cell cycle regulation and cancer biology. This forum will be driven by the publication of specialized and timely research articles, reviews, and commentaries focused on this rapidly moving field–all meant to serve as invaluable tools for cell cycle biologists. The journal will also consider the following article types: book reviews, case reports, hypotheses, methodology articles and short reports.

To allow *Cell Division *to become a significant participant in the Cell Cycle field, we encourage contributions of the most groundbreaking, original and pioneering work from your laboratories. In return, *Cell Division *will provide a quick and impartial peer-review process. Accepted articles will be published online immediately, which will be followed by PubMed listing. To decide whether journal submissions are worthy of in-depth peer-review, manuscripts will be first examined by the Editors-in-Chief. Submissions meeting the journal's standards will then result in appropriate reviews being acquired. Based on reports and scoring provided by the reviewers, the Editors-in-Chief will make a final decision regarding acceptance of the manuscripts. When submitting an article authors are required to complete a declaration of competing interests.

*Cell Division *is published by BioMed Central. Hence, BioMed Central member institutions will not incur *Cell Division *publication charges, and BioMed Central supporter member institutions pay a discounted publication charge. BioMed Central is an independent publishing house committed to ensuring that peer-reviewed biomedical research is open access and immediately and permanently available online without charge or restrictions. The benefits provided by open access to science, medicine and the general public are well documented [3], and we believe an open access journal will be of great benefit to the Cell Cycle field.

We invite you to work together with us to develop this novel, high-visibility forum for the life sciences, the journal *Cell Division*.
